# Antimicrobial Effect of Chitosan Films on Food Spoilage Bacteria

**DOI:** 10.3390/ijms22115839

**Published:** 2021-05-29

**Authors:** Natalia Wrońska, Nadia Katir, Katarzyna Miłowska, Nisrine Hammi, Marta Nowak, Marta Kędzierska, Aicha Anouar, Katarzyna Zawadzka, Maria Bryszewska, Abdelkrim El Kadib, Katarzyna Lisowska

**Affiliations:** 1 Department of Industrial Microbiology and Biotechnology, Faculty of Biology and Environmental Protection, University of Lodz, 12/16 Banacha Street, 90-236 Lodz, Poland; marta.nowak@biol.uni.lodz.pl (M.N.); katarzyna.zawadzka@biol.uni.lodz.pl (K.Z.); 2 Euromed Research Center, Engineering Division (Center Is Part of the Division), Euro-Med University of Fes (UEMF), Route de Meknes, Rond-Point de Bensouda, Fès 30070, Morocco; n.katir@ueuromed.org (N.K.); n.hammi@ueuromed.org (N.H.); a.anouar@ueuromed.org (A.A.); a.elkadib@ueuromed.org (A.E.K.); 3 Department of General Biophysics, Faculty of Biology and Environmental Protection, University of Lodz, 141/143 Pomorska Street, 90-236 Lodz, Poland; katarzyna.milowska@biol.uni.lodz.pl (K.M.); marta.kedzierska@edu.lodz.pl (M.K.); maria.bryszewska@biol.uni.lodz.pl (M.B.)

**Keywords:** chitosan modified films, biodegradable material, antimicrobial activity, cytotoxicity, graphene fillers, metal-oxide clusters

## Abstract

Synthetic materials commonly used in the packaging industry generate a considerable amount of waste each year. Chitosan is a promising feedstock for the production of functional biomaterials. From a biological point of view, chitosan is very attractive for food packaging. The purposes of this study were to evaluate the antibacterial activity of a set of chitosan-metal oxide films and different chitosan-modified graphene (oxide) films against two foodborne pathogens: *Campylobacter jejuni* ATCC 33560 and *Listeria monocytogenes* 19115. Moreover, we wanted to check whether the incorporation of antimicrobial constituents such as TiO_2_, ZnO, Fe_2_O_3_, Ag, and graphene oxide (GO) into the polymer matrices can improve the antibacterial properties of these nanocomposite films. Finally, this research helps elucidate the interactions of these materials with eukaryotic cells. All chitosan-metal oxide films and chitosan-modified graphene (oxide) films displayed improved antibacterial (*C. jejuni* ATCC 33560 and *L. monocytogenes* 19115) properties compared to native chitosan films. The CS-ZnO films had excellent antibacterial activity towards *L. monocytogenes* (90% growth inhibition). Moreover, graphene-based chitosan films caused high inhibition of both tested strains. Chitosan films with graphene (GO, GOP, GOP-HMDS, rGO, GO-HMDS, rGOP), titanium dioxide (CS-TiO_2_ 20:1a, CS-TiO_2_ 20:1b, CS-TiO_2_ 2:1, CS-TiO_2_ 1:1a, CS-TiO_2_ 1:1b) and zinc oxide (CS-ZnO 20:1a, CS-ZnO 20:1b) may be considered as a safe, non-cytotoxic packaging materials in the future.

## 1. Introduction

As a result of technological development, the demand for natural materials with unique physico-chemical properties is increasing. Currently, the synthesis of polymers is a widely studied process, but special attention is sacred to biodegradable, nontoxic materials. These materials should be readily available with low production costs. Chitosan is a promising feedstock for the production of functional biomaterials. Due to the presence of functional groups, it can be subjected to various modifications in order to obtain good biological activity.

Chitosan, a linear β-1,4-D-glucosamine, is a natural polysaccharide obtained from the deacetylation of chitin [1]. It is the second most widespread polysaccharide on Earth, after cellulose. This polymer is commonly obtained from chitin, which composes the exoskeleton of crustaceans (crabs, shrimps, crayfish). Chitosan is also synthesized by fungi, such as *Aspergillus niger* or *Penicillium notatum* [2,3,4]. Total biodegradability and biocompatibility are common features of a broad group of biopolymers, whereas the versatile use of chitosan is mainly due to the presence of amino groups in the polymer backbone. The presence of amino groups in the polymer chain gives some valuable characteristics, e.g., the ability to chelate metals and exhibit catalytic and antimicrobial activities. One line of research is the use of chitosan as a component of food packaging. Increasing consumer demands for safe, unprocessed foods and prolonged storage time has mobilized the food industry to introduce antimicrobial food packaging. Currently, people are more willing to accept natural compounds as preservatives due to their safety [5]. Commonly used metal and plastic packaging are serious environmental problems. They are often enriched with toxic compounds (polyethylene, polypropylene, and other petroleum compounds) that harm human health. In addition to the protective function against environmental factors, the ideal package should also influence the quality and safety of the product. Moreover, advances in the search for new materials capable of extending the shelf life of food products may significantly reduce the current worldwide food waste problem, which is a cause of inefficiency of the global food system and contributes to environmental pollution. Films made from chitosan composites could be an excellent alternative to traditional packaging [6,7]. Food contamination, whose source can be, e.g., packaging processes, poses a severe threat to human health (foodborne infection). Pathogens developing in poorly stored products may cause food poisoning or even debilitating infective diseases such as meningitis [8]. Bacteria that cause food spoilage are *Listeria monocytogenes*, *Staphylococcus aureus*, *Salmonella typhimurium*, and *Campylobacter jejuni*. Several works have described the antimicrobial activity of chitosan [9,10,11,12]. The antibacterial activity of this polymer can be related to the interaction between the amino groups of chitosan and the electronegative charges of the bacterial cell surface, leading to the leakage of intracellular components [13]. In the case of using chitosan in the food industry, it is necessary to obtain stable films. Mineral components may play the role of stabilizers. Our previous works demonstrated that the addition of a low amount of fillers, e.g., graphene and metal oxide, allowed us to obtain stable chitosan nanocomposites with excellent mechanical properties, thermal stability, and antimicrobial activity against *S. aureus* and *E. coli* [14,15]. In this work, we analyzed the effect of selected chitosan films on the bacteria *Listeria monocytogenes* and *Campylobacter jejuni*.

*L. monocytogenes* is a facultative anaerobic gram-positive bacterium and is a major foodborne pathogen [16]. This microorganism occurs in soil, water, decaying vegetation, and animals [17]. Moreover, *L. monocytogenes* can develop at low temperatures. This pathogen can be found in minimally processed products stored under refrigerated conditions, e.g., in vegetables or dairy products. Eating food contaminated with *L. monocytogenes* causes listeriosis. This disease is hazardous for older people with a weakened immune system, pregnant women, and newborns [18]. *Campylobacter jejuni* is a microaerophilic gram-negative bacterium. *Campylobacter sp*. are widespread in nature. The consumption of contaminated poultry products very often causes campylobacteriosis in humans [19]. The main sources of *Campylobacter* infections are poultry, milk, farms, and domestic animals, while the significant reservoirs of this pathogen are breeding farms and birds. These bacteria occur in the gut of many species of birds [20]. Moreover, many studies have shown that *C. jejuni* can survive poultry handling, processing, and packaging [21,22,23]. Currently, finding adequate protection of fresh food products against *L. monocytogenes* and *C. jejuni* is a major challenge for biotechnology.

The purposes of this study were to evaluate the antibacterial activity of a set of chitosan-metal oxide films and different chitosan-modified graphene (oxide) films against two foodborne pathogens: *Campylobacter jejuni* ATCC 33560 and *Listeria monocytogenes* 19115. Moreover, we wanted to check whether the incorporation of antimicrobial constituents such as TiO_2_, ZnO, Fe_2_O_3_, Ag, and graphene oxide (GO) into the polymer matrices can improve the antibacterial and mechanical properties of these nanocomposites. Finally, this research helps elucidate the interactions of these materials with eukaryotic cells.

Metal agents such as zinc oxide, titanium dioxide, and silver have antimicrobial potential, but their properties depend on the size, morphology, and distribution of nanoparticles [24,25]. Recently, metal oxides have attracted attention in food applications because they are stable under high pressure and temperature. Moreover, they may occur in difficult food-processing conditions, and they are also regarded as safe for humans relative to organic substances [26,27]. Food and Drug Administration (FDA) has included zinc oxide nanoparticles in GRAS (generally recognized and safe). Increasing interest has been also recently dedicated to graphene and its derivatives for producing functional nanocomposites. Moreover, graphene has beneficial properties (conductivity, catalytic and adsorptive ability, electronic mobility, conductivity, sensing, and biological activity) that show new avenues in biomedicine and wearable electronics [14,28,29]. Additionally, titanium dioxide is an attractive photocatalyst because it is inexpensive, chemically stable, nontoxic and generally recognized as safe (GRAS) [30,31]. This study verifies their potential use in the future as alternatives to currently used materials.

## 2. Results and Discussion

### 2.1. Antimicrobial Activity

Regarding the use of modified chitosan films as food packaging material, the microorganisms that most often cause spoilage of food products were selected for this study.

The antimicrobial activity of chitosan-clustered metal oxide films (CS, CS-ZnO, CS-TiO_2_, CS-Fe_2_O_3_) and chitosan graphene nanocomposite films was assessed using the bacteria *Listeria monocytogenes* and *Campylobacter jejuni*. The results were related to the control sample—native chitosan film (CS). All tested films were homogenous, not brittle, and had excellent mechanical stability, which is an essential feature in using chitosan films as active bioplastic packaging materials. The characteristics (XRD, DRIFT analysis, SEM, contact angle, thermal and mechanical analysis) of selected films used in the experiments are included in our previous work [14,15]. All tested films are presented in Table 1 and Table 2.

**Figure 1:**
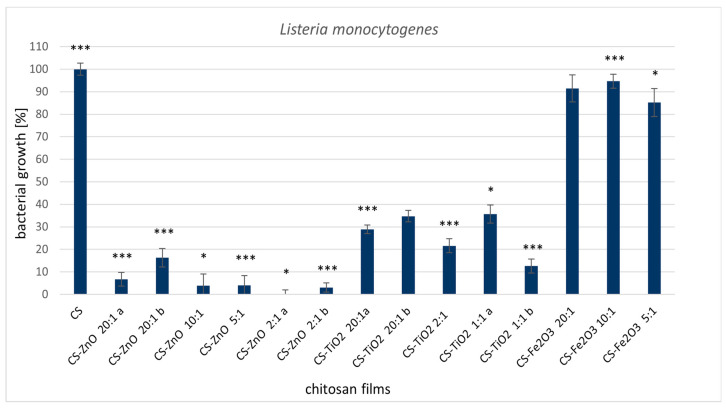
Reduction of *Listeria monocytogenes* ATCC 15313 with treatments with metal-oxide chitosan films after 24 h incubation. The value from control group (CS) was set as 100%. Statistical significance is marked in relation to the chitosan nanocomposite (CS) (*n* = 6, * *p* < 0.05, *** *p* < 0.001).

**Figure 2:**
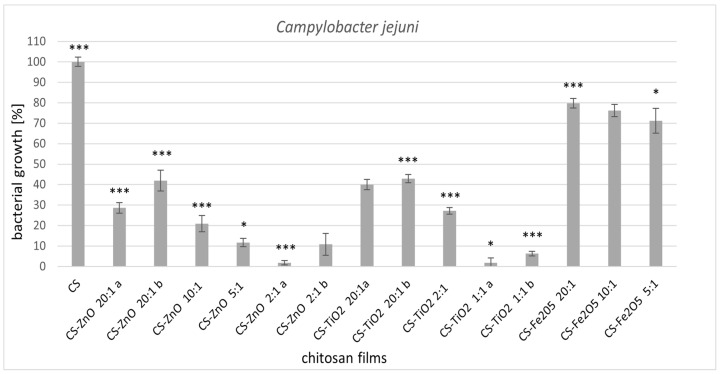
Reduction of *Campylobacter jejuni* ATCC 33560 with treatments with metal-oxide chitosan films after 24 h incubation. The value from control group (CS) was set as 100%. Statistical significance is marked in relation to the chitosan nanocomposite (CS) (*n* = 6, * *p* < 0.05, *** *p* < 0.001).

The analyzed chitosan metal-oxide films clearly exhibited antimicrobial properties against *L. monocytogenes* (Figure 1) and *C. jejuni* (Figure 2). Interestingly, the CS-ZnO films had excellent activity towards gram-positive bacteria (90% growth inhibition). For the CS-ZnO 2:1a sample, a bactericidal effect was noted (Figure 1). In our previous work, CS-ZnO films also showed the best activity against gram-positive *Staphylococcus aureus* strains [15]. It has been shown that zinc oxide particles display great antibacterial activity compared to other metals [32,33]. It has been proven that smaller ZnO particles have better antibacterial activity [34,35]. Several mechanisms of ZnO antimicrobial activity have been suggested: the first mechanism include the release of antibacterial ions [36], the second consist in the formation of ROS (by the effect of light radiation) and the third describes the interaction of zinc particles with microorganisms [37]. Moreover, these nanoparticles may reduce the attachment of microbes to different surfaces [38,39]. Zinc oxide nanoparticles containing polyester surfaces showed high antibacterial activity against *S. aureus* and *E. coli* [40]. This action of zinc oxide repels or inhibits the initial step of bacterial adhesion [41,42]. Rhaman et al. [43] also showed that the antimicrobial activity of chitosan films was significantly improved when zinc oxide nanoparticles were incorporated. Ejaz et al. [44] prepared a film incorporating zinc oxide nanorods and clove essential oil with bovine skin gelatine as the polymer matrix. This combination showed maximum antibacterial activity against *L. monocytogenes* and *S. typhimurium* inoculated in shrimp during refrigerated storage. Cellulose-chitosan films containing monolaurin have an inhibitory effect on *L. monocytogenes* in ultrafiltered cheese [45]. Jovanovic et al. [46] tested chitosan-gelatine films with thyme essential oil. This film has activity towards *L. monocytogenes* 19115 and 19112.

Chitosan-zinc oxide films also showed great antibacterial activity against *C. jejuni*. More abundant bacterial growth was observed in films with lower zinc content. However, in chitosan films where the addition of zinc oxide was in the ratio CS:ZnO (5:1 and 2:1), the bacterial growth inhibition was 90%. Xie et al. [47] described that *C. jejuni* was sensitive to zinc oxide nanoparticles. Moreover, they obtained a bactericidal effect in these studies. *Campylobacter* spp. were the bacteria most sensitive to chitosan [48]. In our research, pure chitosan film caused a very slight inhibition of *C. jejuni* growth. Perhaps the difference was due to the applied form of chitosan.

The other samples supplemented with titanium dioxide also showed great antimicrobial activity, inhibiting the growth of *L. monocytogenes* by 65–88%. Nanocomposites of chitosan and nanosized titanium dioxide have been described by Siripatrawas and Kaewklin [30]. The results suggested that chitosan films containing 1% TiO_2_ exhibited antimicrobial activity against *S. aureus* and *E. coli* (50% growth inhibition) and *S. typhimurium* and *P. aeruginosa* (20% growth inhibition). Xing et al. [49] also showed that chitosan-TiO_2_ nanocomposites have an inhibitory effect on the growth of *S. aureus* and *E. coli*. Many articles described antibacterial activities of visible light-responsive TiO_2_ photocatalyst [50,51]. Cheng et al. [51] reported the antibacterial potential of visible light-irradiated C-doped TiO_2_ on *S. aureus*, *A. baumannii* and *S. flexneri*. They observed that photocatalysis was effective against tested bacteria [51]. Other studies indicate a possible photocatalytic bactericidal effect against *E. coli* strain using silver modified TiO_2_ [52]. It is suggested that photocatalytic antibacterial mechanism of TiO_2_ initially damages the bacterial cell surfaces [53]. Further, the leakage of intracellular components is observed and finally the photocatalysis destroys the cell debris [54]. Yadaw et al. [55] tested the anatase titanium dioxide nanoparticles with copper. They observed bactericidal effect of Cu3-TiO_2_ nanoparticles after 240 min. irradiation. They also showed that incubation of Cu-TiO_2_ nanoparticles with bacteria in the dark did not affect the survival of bacteria.

A weaker antimicrobial effect was observed for all chitosan iron oxide films, where abundant bacterial growth was detected. Iron oxide nanoparticles have reduced the growth of *L. monocytogenes* in a concentration-dependent manner [56]. Arakha et al. [57] showed that iron oxide nanoparticles have insignificant antibacterial activity against *B. subtilis* and *E. coli*. However, coating with chitosan improved their antimicrobial activity.

The influence of the amount of incorporated metal oxide is especially evident in *Campylobacter jejuni* (Figure 2) for all tested chitosan-clustered metal oxide films. The biological activity increased by increasing the number of metal components in the CS film. The most significant bacterial growth inhibition was reported for CS-ZnO 2:1a and CS-TiO_2_ 1:1 (95% inhibition), with the highest molar ratio of metal. As in the *L. monocytogenes* strain, *C. jejuni* was also the least sensitive to chitosan films of iron oxide (20% bacterial growth inhibition).

**Figure 3:**
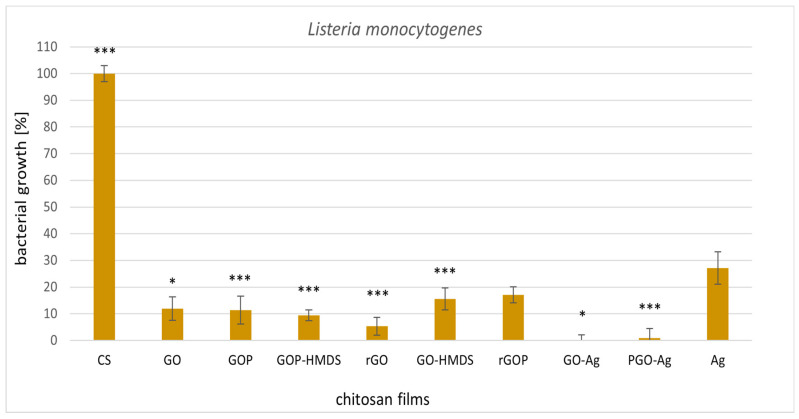
Reduction of *Listeria monocytogenes* ATCC 15313 with treatments with metal-oxide chitosan films after 24 h incubation. The value from control group (CS) was set as 100%. Statistical significance is marked in relation to the chitosan nanocomposite (CS) (*n* = 6, * *p* < 0.05, *** *p* < 0.001).

**Figure 4:**
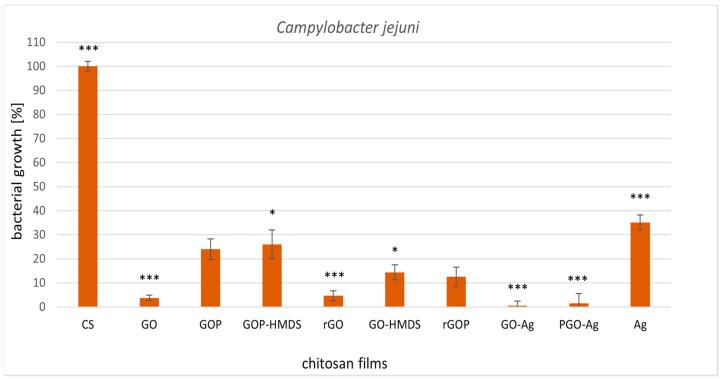
Reduction of *Campylobacter jejuni* ATCC 33560 with treatments with metal-oxide chitosan films after 24 h incubation. The value from control group (CS) was set as 100%. Statistical significance is marked in relation to the chitosan nanocomposite (CS) (*n* = 6, * *p* < 0.05, *** *p* < 0.001).

Subsequently, we examined the biological activity of graphene-based chitosan nanocomposites and silver nanoparticles loaded in chitosan film (Figure 3 and Figure 4). All chitosan films supplemented with graphene caused high inhibition of the tested microorganisms compared to native chitosan film. 100% inhibition of *L. monocytogenes* growth was observed for the graphene-silver chitosan film (GO-Ag) (Figure 3). The other graphene films showed satisfactory results (80–90% growth inhibition) of both tested strains. The least active sample in relation to the tested strains was chitosan-silver nanocomposites. The influence of chitosan-silver films on the microorganisms tested in this work has not been described thus far. Morsy et al. [58] found that pullulan-edible packaging with silver nanoparticles inhibited *L. monocytogenes* and *S. aureus* in vacuum packaged ready-to-eat turkey deli meat stored in refrigerated conditions. In our study, graphene-silver chitosan films (GO-Ag and PGO-Ag) had the best antibacterial activity against the tested strains. We suppose that the addition of graphene oxide or phosphorylated graphene oxide enhances the activity of this material. Mazaheri et al. [59] reported the antimicrobial activity of chitosan-graphene nanocomposites against the gram-positive strain *S. aureus*. Great antibacterial activity towards methicillin-resistant *S. aureus* and *E. coli* was displayed by chitosan-iron oxide-coated graphene hydrogel films [60].

#### 2.1.1. Permeability of Bacterial Cell Membranes

**Figure 5:**
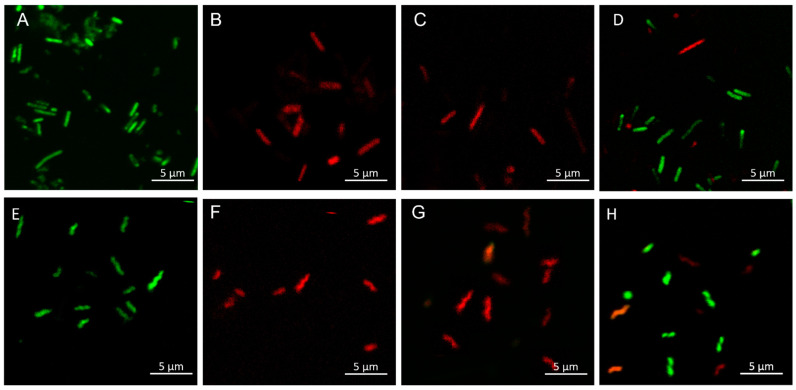
Permeability of the L. *monocytogenes* (**A**–**D**) and C. *jejuni* (**E**–**H**) cell membranes after treatment with chitosan films. (**A**)—control, (**B**)—CS-ZnO 2:1a, (**C**)—CS-GO-Ag, (**D**)—CS-Ag (**E**)—control, (**F**)—CS-ZnO 2:1a, (**G**)—CS-GO-Ag, (**H**)—CS-Ag.

Tested samples with the best antibacterial activity towards *L. monocytogenes and C. jejuni* were stained using the LIVE/DEAD BacLight^TM^ Bacterial Viability Kit according to the manufacturer’s protocol. Syto 9 penetrates cells with damaged and intact membranes, while PI penetrates only the cells with a damaged membrane and reduces the Syto 9 dye. Bacterial cells with intact membranes were stained green (Figure 5A,D,E,H), while dead or injured cells were stained red (Figure 5B–D,F–H). However, it was difficult to obtain legible pictures of chitosan film samples by fluorescence confocal microscopy. In this case, scanning electron microscopy (SEM) proved to be a suitable technique.

The results confirm the strong antibacterial properties of the samples (CS-ZnO, CS-GO-Ag) because all microorganisms per microscope field were stained red by PI, excluding the control (bacteria incubated on pure chitosan film). Figure 5D,H shows the bacteria (*L. monocytogenes*—Figure 5D and *C. jejuni*—Figure 5H) after incubation with less effective material (CS-Ag).

It is known that silver and zinc oxide nanoparticles affect bacterial membranes [61], and the antimicrobial mechanism of action of Zn nanoparticles is based on their stability to induce oxidative stress in bacterial cells [62]. Zinc ions may interact with the thiol group of bacterial respiratory enzymes, stimulating the production of ROS. As a consequence of this action, bacterial cell membranes, DNA, and mitochondria are damaged, leading to cell death. [63,64,65]. Silver nanoparticles are effective as antibacterial agents at low concentrations [66], and they can interact with cell membrane proteins and bacterial DNA, which contain phosphorus and sulfur complexes that have a high attraction to Ag nanoparticles [67]. Based on the results, we found that a better antibacterial effect was obtained in the synergistic action of silver nanoparticles and graphene oxide in the chitosan matrix.

The mechanism of action of graphene-based chitosan films may be caused by direct contact and interaction of the graphene sharp nanosheets with the bacterial cell membranes, resulting in the alteration of membrane permeability [14].

#### 2.1.2. Morphological Changes of *L. monocytogenes* and *C. jejuni* Cells Visualized by Scanning Electron Microscopy (SEM)

**Figure 6:**
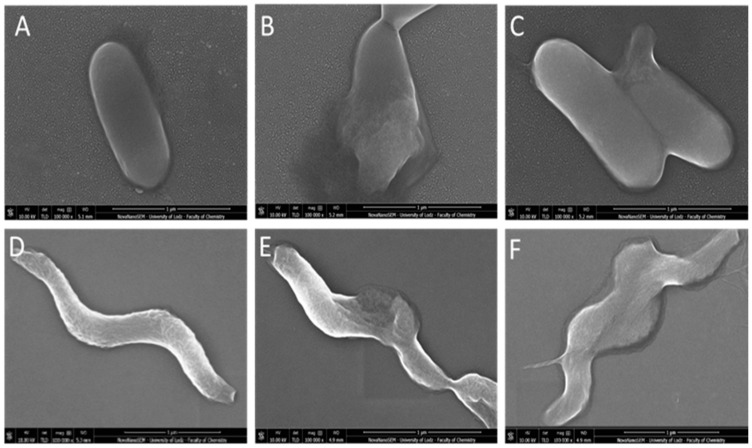
SEM images of *L. monocytogenes* (**A**–**C**) and *C. jejuni* (**D**–**F**) cells after treatment with chitosan films. (**A**)—control, (**B**)—CS-ZnO 2:1a, (**C**)—CS-GO-Ag, (**D**)—control, (**E**)—CS-ZnO 2:1a, (**F**)—CS-GO-Ag.

In this part of the study, we focused on chitosan-zinc oxide composites (CS-ZnO 2:1a) and chitosan-graphene composites with silver (CS-GO-Ag). The selected samples revealed the highest antibacterial activity. We tried to show the changes in cell morphology and structure. Figure 5 displays representative SEM images obtained for *L. monocytogenes* and *C. jejuni* after chitosan film treatment (pure CS film, CS-ZnO 2:1a, CS-GO-Ag). For *L. monocytogenes*, untreated bacteria appeared as an intact rod-shaped form (Figure 5A) without evidence of cell wall collapse or rupture. In contrast, after CS-ZnO 2:1 or CS-GO-Ag (Figure 6B,C) exposure, bacterial cells showed cell wall damage and leaked out of their cellular components.

In untreated *C. jejuni* cells (Figure 5D), the structure of helical-shaped bacteria retained the correct form. After incubation with CS-ZnO 2:1 or CS-GO-Ag films, the cell morphology was substantially changed. The cells become partly deformed (Figure 6E,F).

Observations made by confocal microscopy and scanning electron microscopy were consistent, both showing morphological and structural changes. In both cases, antibacterial action was related to cell fluid leakage due to cell morphology destruction. However, the mode of antimicrobial action of chitosan films with nanosized fillers is not a simple mechanism but a complicated process that still needs more research and clarifications.

### 2.2. Cytotoxicity of Films

**Figure 7:**
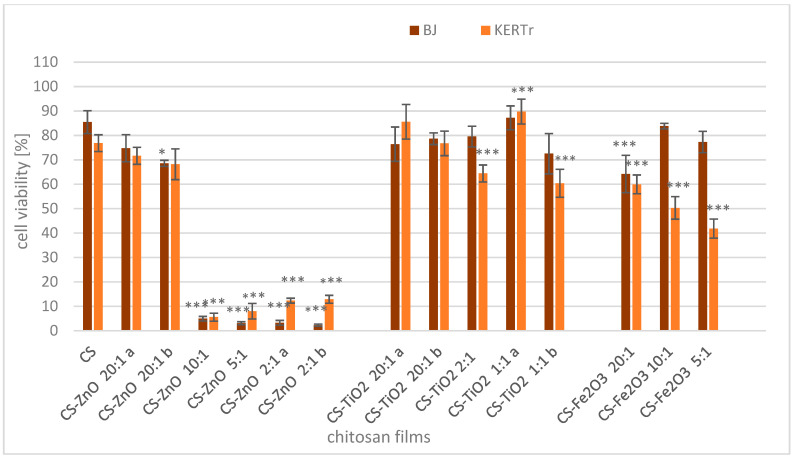
Viability of human fibroblast (BJ) and keratinocyte (KERTr) cells after 24 h of exposure to chitosan and chitosan−metal oxide composites. Cell viability was calculated as the percent ratio of absorbance of the samples to the referent control (untreated cells). The value from control group was set as 100%. Statistical significance is marked in relation to the chitosan nanocomposite (CS) (*n* = 6, * *p* < 0.05, *** *p* < 0.001).

**Figure 8:**
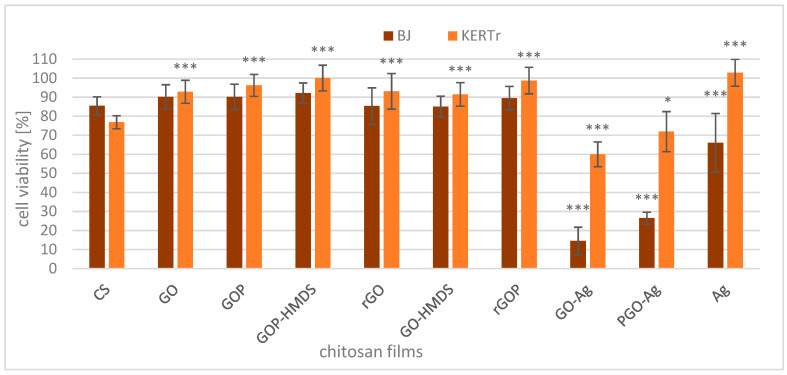
Viability of human fibroblast (BJ) and keratinocyte (KERTr) cells treated with chitosan and chitosan-graphene composites for 24 h. Cell viability was calculated as the percent ratio of absorbance of the samples to the referent control (untreated cells). The value from control group was set as 100%. Statistical significance is marked in relation to the chitosan nanocomposite (CS) (*n* = 6, * *p* < 0.05, *** *p* < 0.001).

The application of nanomaterial technology is rapidly expanding. People can be exposed to skin contact with nanomaterials; therefore, it is essential to assess the toxicity of all new composites to human skin cells. The effects of chitosan-metal oxide and chitosan-graphene materials on the viability of human skin cells (fibroblasts BJ and keratinocytes KERTr) were assessed by MTT testing. The percentage of viable cells was computed relative to controls (cells incubated without biocomposite), whose viability was considered 100%. The obtained results are presented in Figure 7 and Figure 8.

After incubating skin cells with unmodified chitosan film (CS), cell viability slightly decreased to 85% for BJ cells and 77% for KERTr (Figure 7). Chitosan materials modified with zinc oxide at a ratio of 20:1 (CS-ZnO 20:1a and CS-ZnO 20:1b) only slightly reduced the viability of BJ and KERTr concerning the unmodified form (CS) and only in one case. These changes were statistically significant, while the composites with a higher zinc oxide content (CS-ZnO 10:1–CS-ZnO 2:1) were very toxic to both tested cells. After 24 h of incubation with these films, the cell viabilities were less than 13%. The presence of zinc oxide is crucial in the toxicity of the films tested. The results show the concentration-dependent cytotoxic effect of chitosan-zinc oxide composites on skin cells. In the films with low ZnO content, the decrease in viability was minimal, while at higher concentrations of oxide, the viability decreased drastically. The toxicity of these films is probably due to the effect of zinc oxide on the cell membrane and the production of reactive oxygen species (ROS). The literature available on ZnO nanomaterials suggests that the production of reactive oxygen species and induced inflammatory responses are factors that govern the toxicity of ZnO nanoparticles [68,69]. Several researchers observed increased lipid peroxides in the cells, giving rise to more free radicals and damaged biomolecules such as DNA and other biostructures in the cell. They observed dose- and exposure time-dependent manners, where higher concentrations and prolonged exposure induced cell death with the loss of cell metabolism and membrane integrity [70,71,72].

For chitosan modified with titanium oxide and BJ cells, no significant changes compared to CS were observed. Similar results were obtained after incubation of the BJ cell line with chitosan-iron oxide films (CS-Fe_2_O_3_ 10:1 and CS-Fe_2_O_3_ 5:1). Only CS-Fe_2_O_3_ 20:1 decreased BJ cell viability to approx. 64%. The viability of the KERTr cells incubated with CS-TiO_2_ 1:1a was slightly higher than that incubated with CS, but CS-TiO_2_ 1:1b and CS-TiO_2_ 2:1 caused a decrease in cell viability. Additionally, CS-Fe_2_O_3_ films were more toxic to keratinocytes than unmodified chitosan. The results prove the typical concentration-dependent cytotoxic effect of CS-Fe_2_O_3_ films on KERTr cells. Keratinocytes were more sensitive than fibroblasts to the action of films modified with iron oxide (Figure 7).

The cytotoxicity of chitosan membranes with the addition of iron oxide, similar to chitosan-zinc oxide, is due to the production of ROS and damage to cell membranes. Previous studies reported nanoparticles with iron oxide-induced oxidative stress, genotoxicity, and apoptosis in mammalian cells [73,74,75].

The presence of titanium dioxide in chitosan films does not cause significant changes in film toxicity to fibroblasts. The toxicity of TiO_2_ nanoparticles towards human skin cells is unclear. These results are in agreement with previous in vitro studies [76]. Browning et al. [76] showed that TiO_2_ nanoparticles could penetrate the cytoplasm and nucleus of human skin fibroblast cells after 24 h of exposure, but they did not observe chromosomal aberrations or cytotoxicity of these nanoparticles. Kiss et al. [77] reported that TiO_2_, when exposed directly to cell cultures in vitro, exerts significant and cell type-dependent effects on the viability, proliferation, apoptosis, and differentiation of skin cells. Using various nuclear microscopy methods, they also demonstrated that TiO_2_ nanoparticles in vivo do not penetrate the intact epidermal barrier. In this work, changes in keratinocyte viability were detected for films containing higher concentrations of TiO_2_. However, these changes also depend on the method of synthesis of the film. Two films, CS-TiO_2_ 1:1a and CS-TiO_2_ 1:1b contain the same concentrations of titanium dioxide, but the results are different. The difference in cell toxicity could be due to a trapped solvent in which the synthesis was performed.

The chitosan-graphene composites without silver show slightly lower cytotoxicity compared to CS (Figure 8). These results are statistically significant for KERTr cells. It follows that the presence of graphene oxide and its derivatives in the chitosan films makes them less toxic to skin cells, which is advantageous in terms of the practical application of these materials. Qiao et al. [78] showed that graphene oxide (GO) protects normal cells from oxidative damage by removing free radicals generated by X-ray radiation. GO at high concentrations (100 and 500 μg/mL) causes cell death and DNA damage but can effectively remove ROS at a concentration of 10 μg/mL. Thus, low concentrations of GO can be used as an effective protective agent in occupational and therapeutic settings. However, it should be noted that the mechanism of action in suspension may be fundamentally different from the mechanism of action occurring in hydrogels or solid-state films.

After adding chitosan-graphene composites with silver (GO-Ag and PGO-Ag), the viability of cells significantly decreased. The BJ cells were more sensitive than KERTr cells to the action of chitosan-graphene-silver materials, meaning the toxicity of various composites also depends on the type of cells and their sensitivity. The results were expected because several studies demonstrated the cytotoxicity of AgNPs towards different types of cells, such as neuroendocrine cells [79], human peripheral blood mononuclear cells [80], lung epithelial cells [81], and many others, interfering with their cellular functions and causing DNA damage and apoptosis.

However, the viability of the KERTr cells treated with chitosan-silver composites (without graphene) was higher than that of cells treated with chitosan alone or chitosan-graphene-silver films. The mechanism of this action is unknown, but silver ions are released in chitosan films with graphene and silver, which are toxic to cells. On the other hand, in a chitosan film without graphene, silver is more strongly bound to the chitosan structure and is not released or released less, making it nontoxic to KERTr cells and less toxic to BJ cells than GO-Ag and PGO-Ag. It should also be noted that these cells were incubated in other cellular media, so the environment may also play a role in the release of silver from composites.

## 3. Materials and Methods

### 3.1. Materials

Chitosan of medium molecular weight and 85% deacetylation degree, titanium diisopropoxide bis(acetylacetanate) (Ti(acac)_2_OiPr_2_), iron (III) acetylacetonate (Fe(acac)_3_), zinc acetate (Zn(OAc)_2_) and 3-(4,5-dimethylthiazol-2-yl)-2-5-diphenyltetrazolium bromide (MTT) were purchased from Sigma-Aldrich (Hamburg, Germany). The human fibroblast BJ (CRL-2522) cell line and the human keratinocyte CCD 1102 KERTr (CRL-2310) were purchased from American Type Culture Collection (ATCC^®^, Manassas, VA, USA). Keratinocyte serum-free medium with added keratinocyte supplements, including bovine pituitary extract (BPE), human recombinant epidermal growth factor (EGF), fetal bovine serum (FBS), and Dulbecco’s modified Eagle’s medium (DMEM), was purchased from Gibco, Thermo Fisher Scientific (Waltham, MA, USA). Graphite flakes, potassium permanganate, sodium nitrate, sulfuric acid, hydrochloric acid, hydrazine, hydrogen peroxide, phosphoryl chloride, bis-trimethylsilylamine, ethanol, tetrahydrofuran, and acetic acid were obtained from Across and Sigma-Aldrich (Darmstadt, Germany). Phosphate-buffered saline (PBS) was procured from BioShop (Burlington, VT, Canada). Glutaraldehyde 25% and osmium tetroxide 4% solution were acquired from Agar Scientific (Stansted, UK). Absolute ethanol was bought from EMSURE (Darmstadt, Germany). The LIVE/DEADTM BacLightTM Bacterial Viability Kit was attained from Thermo Fisher Scientific (Warsaw, Poland).

### 3.2. Preparation of Chitosan-Metal Oxide Films

**Figure 9:**
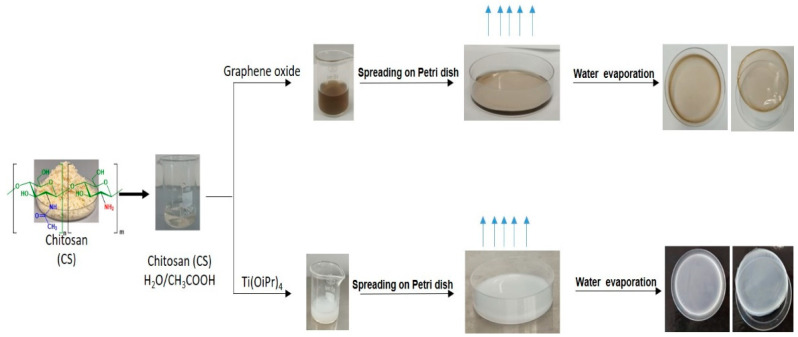
Preparation scheme of chitosan-clustered metal oxide films and chitosan graphene nanocomposite films.

First, 0.05 g of chitosan was dissolved in 4 mL of acetic acid solution. A given mass of the metal precursor with an NH2:M molar ratio of (1:1; 2:1; 5:1; 10:1; 20:1) was added to the abovementioned solution. The mixture was then stirred for 1 h at room temperature to obtain a homogeneous dispersion, and the resulting solution was cast onto a clean Petri dish for 24 h until total evaporation of the solvent (Figure 9). Data from SEM, EDX and FTIR analysis are available in the supplementary materials (Figures S1–S3, Supplementary Materials).

### 3.3. Preparation of Functionalized Graphene Oxide Fillers

Graphene oxide (GO) was obtained from graphite flakes using a modified hummers method [82]. GOP, *rGO* and *HMDS-GO* were prepared according to the literature procedures [14,83]. *rGOP*: Hydrazine (0.3 mL) was added to a dispersion of GOP (16 mg) in 40 mL H_2_O. The mixture was heated at 60 °C for 24 h. After filtration and extensive washing of the precipitate with ethanol, the collected solids were dried at 60 °C for 12 h, giving rise to rGOP.

*HMDS-GOP*. Bis-trimethylsilylamine (76 mmol) was added to a suspension of GOP (40 mg in 100 mL of toluene). The mixture was magnetically stirred for 24 h at 80 °C. The powder was recovered by filtration, washed with toluene, and dried for 6 h in an oven at 60 °C.

### 3.4. Preparation of Chitosan-Graphene Films

**Figure 10:**
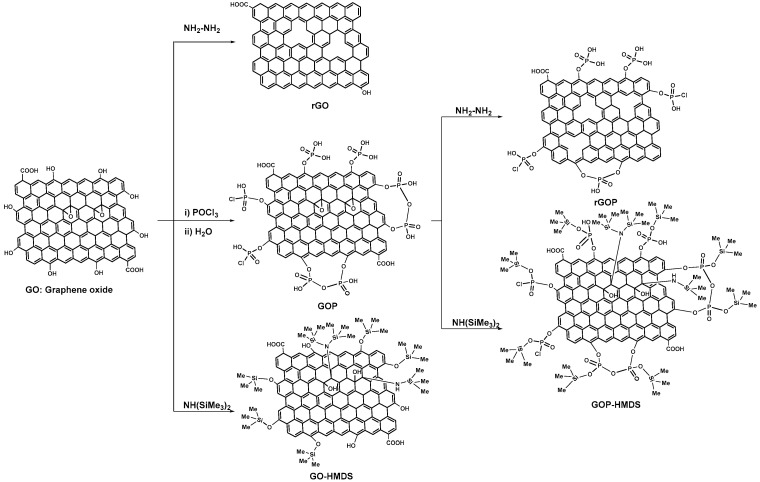
Scheme illustrating the synthesis of functionalized graphene fillers (GOP, rGOP, rGO, GO-HMDS and GOP-HMDS).

The procedure used to prepare CS-GO, CS-GOP, CS-rGO, CS-rGOP, CS-GO-HMDS, or CS-GOP-HMDS films (Figure 10) is similar to a previous study [14]. Briefly, 50 mg of chitosan was dissolved in 4 mL of 1% (*v*/*v*) acetic acid solution and kept under vigorous stirring for 120 min. Then, 1.5 mg of GO, GOP, rGO, or rGOP, GO-HMDS, or GOP-HMDS was dispersed in 2 mL of the 1% (*v*/*v*) acetic acid solution and submitted to sonication for 90 min. The suspension was gradually added to the chitosan solution, and the resulting mixture was stirred for an additional 90 min. The resulting solution was finally poured into plastic Petri dishes and dried at room temperature to form films.

CS-Ag: First, 50 mg of CS was dissolved in 7 mL of 1% (*v*/*v*) acetic acid solution. Then, 1.5 mg of AgNO_3_ was added to the solution, and the mixture was stirred for 3 h before being poured into plastic Petri dishes and dried at room temperature to form films.

CS-GO-Ag/CS-GOP-Ag: First, 1.5 mg of GO or GOP dispersed in a 2 mL of 1% (*v*/*v*) acetic acid solution and submitted to sonication. Then, 1.5 mg of AgNO_3_ was added to the GO or GOP suspension, followed by the addition of 5 mL of 1% (*v*/*v*) acetic acid solution. The mixture was stirred for one minute before the addition of 50 mg of CS. The suspension was stirred for 2 h, poured into plastic Petri dishes and dried at RT to form films.

### 3.5. Determination of Antimicrobial Activity

*Listeria monocytogenes* ATCC 15313 and *Campylobacter jejuni* ATCC 33560 were obtained from American Type Culture Collection (ATCC) (Wesel, Germany) and were grown on Brucella agar broth (Becton Dickinson GmbH, Heidelberg, Germany) supplemented with 5% (vol/vol) horse serum (Sigma-Aldrich, Hamburg, Germany) and Columbia agar with 5% sheep blood (Becton Dickinson GmbH, Heidelberg, Germany). *Listeria monocytogenes* were grown for 48 h, followed by a subculture for 24 h at 37 °C. *Campylobacter jejuni* was developed for 48 h in addition to a subculture for 24 h under microaerophilic conditions.

The antimicrobial activity of modified chitosan films against the tested bacteria was evaluated using Japanese Industrial Standards Z 2801:2000 with modification. The prepared inoculums were centrifuged at 8000 rpm for 10 min and washed twice with phosphate buffer. Then, a bacterial suspension (*L. monocytogenes* or *C. jejuni*) containing 1 × 10^8^ colony forming units (CFU per mL) in a 500-fold diluted Brucella broth medium was prepared.

Next, the bacterial suspension was transferred to chitosan films of 2 cm × 2 cm. Native chitosan films were examined as control samples. After dripping the suspension of *L. monocytogenes* or *C. jejuni* on the films, each sample was covered with a sterile film (1.7 cm × 1.7 cm). The samples were incubated in the moist chamber in the dark for 24 h at 37 °C (*L. monocytogenes*) or at 41 °C under microaerophilic conditions (*C. jejuni*). Next, they were placed in aseptic Falcon tubes containing phosphate buffer, vortexed, and removed from the Falcon tubes. A serial dilution was performed with the remaining solution in phosphate buffer. Out of each dilution, 100 µL of bacterial suspension was seeded on Columbia agar with a 5% sheep blood plate and incubated for 24 h at 37 °C (*L. monocytogenes*) or 41 °C under microaerophilic conditions (*C. jejuni*). After incubation, viable cells of the tested bacteria were counted.

Each type of tested film was examined in triplicate and analyzed individually in four independent experiments. The antimicrobial activity of the tested films was calculated as the percentage of bacterial growth inhibition (SD) towards native chitosan control films.

### 3.6. Confocal Microscopy

Cell visualization was conducted in the Laboratory of Microscopic Imaging and Specialized Biological Techniques (Faculty of Biology and Environmental Protection, University of Lodz) using a Leica TCS SP8 microscope equipped with achromatic plan objectives (Leica) and with magnifications of 63× and 100× (oil immersion).

Tested bacterial cells (*L. monocytogenes, C. jejuni*) were stained using the LIVE/DEAD BacLight^TM^ Bacterial Viability Kit according to the manufacturer’s protocol. The bacterial cells treated and untreated with chitosan film and modified chitosan films were centrifuged at 10,000 rpm for 5 min and washed three times with PBS. Then, the cells were suspended in 100 µl PBS with the addition of 1 µl of the Syto 9 and propidium iodide mix (*v*/*v*:1/1). Next, the bacterial suspension (*L. monocytogenes, C. jejuni*) was vortexed and incubated at 37 °C for 15 min in the dark. Finally, the fluorescence of Syto 9 and propidium iodide was measured at excitation/emission (ex/em) maxima of 480/500 nm and 490/635 nm, respectively.

### 3.7. Scanning Electron Microscopy (SEM)

*Listeria monocytogenes* or *Campylobacter jejuni* cells incubated with suitable/tested chitosan films were washed with phosphate-buffered saline (PBS) and vortexed 3 min. Next, the cells were washed three times with PBS and centrifuged at 10000 rpm for 5 min. Bacterial cells were suspended in a solution of glutaraldehyde and incubated for 16 h at 4 °C. Next, bacterial cells were centrifuged at 10,000 rpm for 5 min and washed three times with PBS. Subsequently, bacterial cells were centrifuged and washed three times in PBS and dehydrated in ethanol solutions (25, 50, 75, 90, and 100%) for 10 min each. The cells were spread on a silicon wafer, dried at 22 °C, and sputtered with a gold layer at 2 nm thickness. SEM images of tested bacterial cells (*L. monocytogenes* or *C. jejuni*) were visualized using a Nova NanoSEM 450 scanning electron microscope (Hillsboro, OR, USA). SEM analyses were performed in immersion mode using a through-lens detector (TLD) at a magnification of 80000×.

### 3.8. Cell Culture

Human fibroblast (BJ) cells were grown as a monolayer in DMEM supplemented with 10% fetal bovine serum (FBS) and 1% streptomycin. The cultures were incubated at 37 °C in an atmosphere of 5% CO_2_. Cells were split for subcultures every two days.

Human keratinocyte (KERTr) cells were grown in keratinocyte serum-free medium Gibco 1705-042^®^ (Gibco, Thermo Fisher Scientific Inc., Waltham, MA, USA) supplemented with keratinocyte supplements (Gibco 3700-015^®^), including bovine pituitary extract BPE (Gibco, Thermo Fisher Scientific Inc., Waltham, MA, USA) and human recombinant epidermal growth factor EGF (Gibco, Thermo Fisher Scientific Inc., Waltham, MA, USA) supplemented with an additional 30 ng/mL human recombinant epidermal growth factor (EGF). The cultures were incubated at 37 °C in an atmosphere of 5% CO_2_. Cells were split for subcultures every two days.

### 3.9. Cytotoxicity Assay

The cytotoxicity of chitosan-metal oxide and chitosan-graphene materials was evaluated by MTT assay. This assay is based on the cellular reduction (by mitochondrial dehydrogenases) of the soluble yellow dye MTT to water-insoluble purple formazan in living cells. Therefore, the amount of formazan crystals is proportional to the number of living cells because the dehydrogenases are inactive in dead cells [84].

The cells were seeded in flat-bottom 24-well plates at a density of 5 × 10^4^ (BJ) and 10 × 10^4^ (KERTr) cells in 400 μL of medium per well. They were treated with films (squares of 0.5 cm × 0.5 cm) and incubated for 24 h. Next, the films were removed, and 200 μL of 0.5 mg/mL MTT solution was added to each well and incubated for 3 h. After this time, the MTT-containing medium was detached, 400 μL of DMSO was added to each well to dissolve formazan crystals, and the absorbance was measured at 570 nm using a microplate spectrophotometer (BioTek, Synergy HTX multimode reader, Winooski, VT, USA). Cell viability was calculated as the percent ratio of absorbance of the samples to the reference control.

### 3.10. Statistical Analysis

Data are presented as mean ± SD from six sets of measurements. The statistical differences between the control and treated groups and differences between films were analyzed by one-way ANOVA, followed by Tuckey’s analysis. *p* < 0.05 was accepted as statistically significant.

## 4. Conclusions

Synthetic materials commonly used in the packaging industry generate a considerable amount of waste each year. Industrial production of plastics requires a substantial number of petroleum-based polymers, which has a negative and extremely worrying impact on public health and the environment (plastics produce many eternal chemical wastes, e.g., microplastics). Therefore, the search for new biological materials to find sustainable alternatives is an urgent task of science. The most useful nanocomposites should have good antimicrobial activity with low cytotoxicity. Herein, we report new chitosan-based films with excellent biological activity. In particular, we showed the highest antibacterial activity of graphene-based chitosan films against *Listeria monocytogenes* ATCC 19115 and *Campylobacter jejuni* ATCC 33560 strains compared to neat chitosan films. Additionally, all tested CS-ZnO films caused growth inhibition of *L. monocytogenes*. In summary, our results provide information on antimicrobial, non-cytotoxic metal-oxide (CS-TiO_2_ 20:1a, CS-TiO_2_ 20:1b, CS-TiO_2_ 2:1, CS-TiO_2_ 1:1a, CS-TiO_2_ 1:1b, CS-ZnO 20:1a, CS-ZnO 20:1b) and graphene oxide chitosan films that may help to solve the problems of environmental pollution with synthetic packaging materials.

## Figures and Tables

**Table 1:** Chemical composition of CS-MOx-f.

Sample Code	Metal Precursors	Molar Ratio NH_2_:Metal Precursor
CS		
CS-ZnO 1:1	Zinc acetate	1:1
CS-ZnO 2:1	Zinc acetate	2:1
CS-ZnO 5:1	Zinc acetate	5:1
CS-ZnO 10:1	Zinc acetate	10:1
CS-ZnO 20:1 a	Zinc acetate	20:1
CS-ZnO 20:1 b	Zinc chloride	20:1
CS-TiO_2_ 1:1 a	Titanium diisopropoxide bis(acac)	1:1
CS-TiO_2_ 1:1 b	Titanium isopropoxide	1:1
CS-TiO_2_ 2:1	Titanium diisopropoxide bis(acac)	2:1
CS-TiO_2_ 20:1a	Titanium diisopropoxide bis(acac)	20:1
CS-TiO_2_ 20:1 b	Titanium isopropoxide	20:1
CS-Fe_2_O_3_ 5:1	Iron(III) acetylacetonate	5:1
CS-Fe_2_O_3_ 10:1	Iron(III) acetylacetonate	10:1
CS-Fe_2_O_3_ 20:1	Iron(III) acetylacetonate	20:1

**Table 2:** Chemical composition of chitosan-graphene films.

Sample Code	Functionalized Graphene Fillers	Metal Precursors
CS-GO	GO (3 wt%)	-
CS-GOP	GO (3 wt%)	-
CS-GOP-HMDS	GOP-HMDS (3 wt%)	-
CS-rGO	rGO (3 wt%)	-
CS-GO-HMDS	GO-HMDS (3 wt%)	-
CS-rGOP	rGOP (3 wt%)	-
CS-GO-Ag	GO(3 wt%)	Silver nitrate (3%)
CS-GOP-Ag	GOP (3%)	Silver nitrate (3%)
CS-Ag	-	Silver nitrate (3%)

## Data Availability

The data presented in this study are available on request from the corresponding author.

## Supplementary Materials

The following are available online at https://www.mdpi.com/article/10.3390/ijms22115839/s1.
