# Supplementary material for: Antimicrobial Effect of Chitosan Films on Food Spoilage Bacteria
Source: Int J Mol Sci. 2021 May 29;22(11):5839. doi: 10.3390/ijms22115839 (PMC8198402; doi:10.3390/ijms22115839)
Supplement: Supplementary file 1 [file ijms-22-05839-s001.zip › ijms-1221501-supplementary.pdf]

Supporting information

Antimicrobial effect of chitosan films on food spoilage bacteria

Natalia Wrońska [1,*], Nadia Katir [2], Katarzyna Miłowska [3], Nisrine Hammi [2], Marta Nowak [1], Marta Kędzierska [3], Aicha Anouar [2], Katarzyna Zawadzka [1], Maria Bryszewska [3], El Kadib Abdelkrim [2] and Katarzyna Lisowska [1,*]

[1] Department of Industrial Microbiology and Biotechnology, Faculty of Biology and Environmental Protection, University of Lodz, 12/16 Banacha Street, 90-236 Lodz, Poland; katarzyna.lisowska@biol.uni.lodz.pl

[2] Euromed Research Center, Engineering Division, Euro-Med University of Fes (UEMF), Route de Meknes, Rond-point de Bensouda, 30070, Fès, Morocco; a.elkadib@ueuromed.org

[3] Department of General Biophysics, Faculty of Biology and Environmental Protection, University of Lodz, 141/143 Pomorska Street, 90-236 Lodz, Poland; maria.bryszewska@biol.uni.lodz.pl

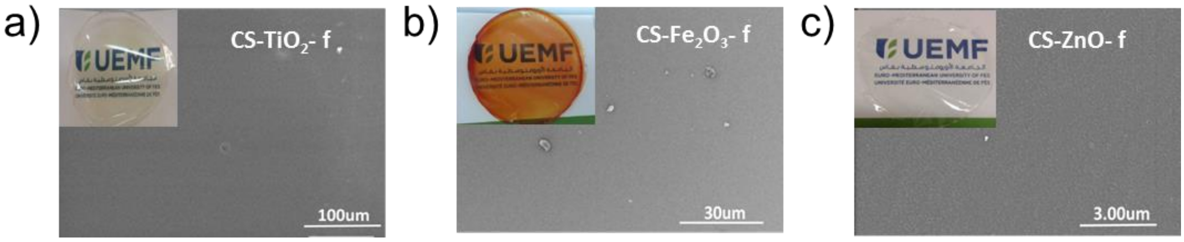

Figure S1: SEM images and digital photographs of the films (inset) of a) CS-TiO_2-f, b) CS-Fe_2O_3-f and c) CS-ZnO-f

The homogeneous distribution of metal ions inside the films was first assessed by scanning electron microscopy (SEM). The digital photographs displayed on the inset of the SEM images as well as the SEM images of the as-synthesized films show the absence of bulky aggregates, phase separation, distinctive zones or rough surfaces, thereby confirming the homogeneity at the microscale.

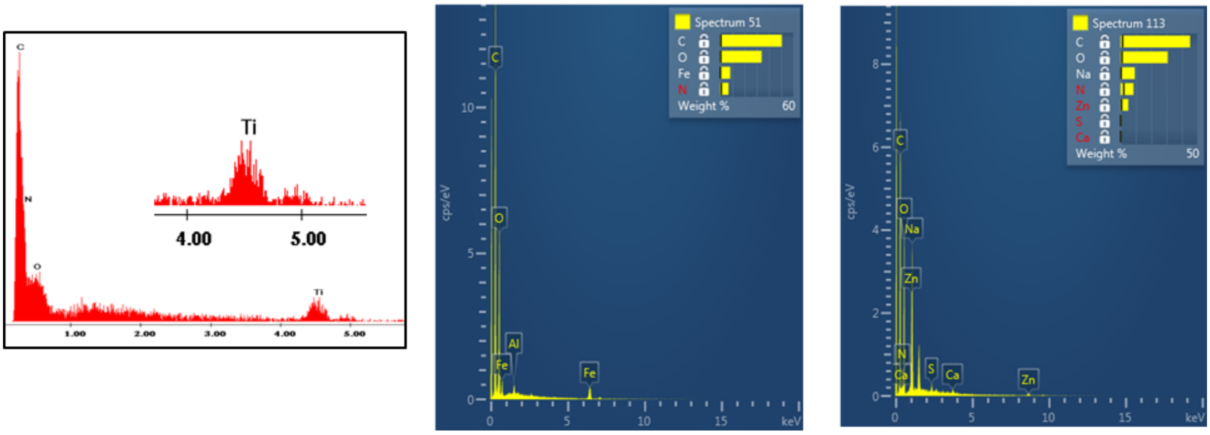

Figure S2: EDX spectra of CS-TiO_2-f in different regions

EDX spectra of CS-TiO_2-f obtained in different regions confirm the presence of the metal element inside the film. In the case of CS-TiO_2-f, the typical titanium signal of Ti was observed at 4.5 keV.

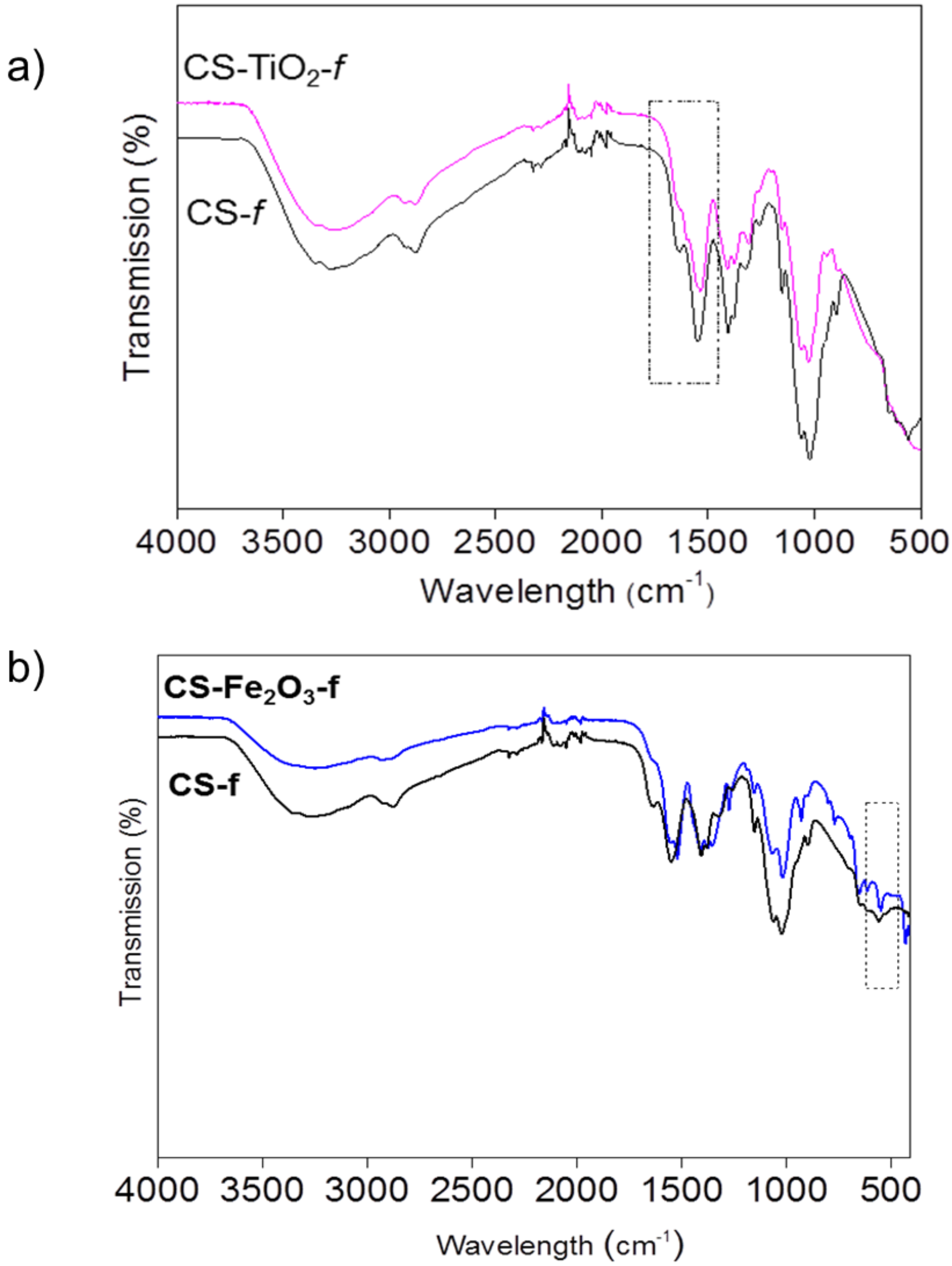

Figure S3: FTIR spectra of a) CS-f and CS-TiO_2-f and b) CS-f and CS-Fe_2O_3-f

The interaction between chitosan and the metal oxide to demonstrate the growth of the metal clusters inside the films was assessed by FTIR. The spectra displayed in figure S3 demonstrate the interaction of glucosamine and N-acetylglucosamine units from chitosan with the metal oxide phase through the shift of the bands as follows:

- In CS-TiO_2-f, there is a blue shift of the adsorption bands of NH_2 (1542cm^{-1}) and $CONH_2$ (1643 cm^{-1}) in the composites compared to the native chitosan, which denotes the N-containing groups bonding with TiO_2;
- In CS-Fe_2O_3-f, a new signal is observed at 552 cm^{-1}, typical of ν(Fe-O) vibration.
